# Role of Charged Residues in the Catalytic Sites of *Escherichia coli* ATP Synthase

**DOI:** 10.4061/2011/785741

**Published:** 2011-07-13

**Authors:** Zulfiqar Ahmad, Florence Okafor, Thomas F. Laughlin

**Affiliations:** ^1^Department of Biology, Alabama A&M University, P.O. Box 610, Normal, AL 35762, USA; ^2^Department of Biological Sciences, East Tennessee State University, Johnson City, TN 37614, USA

## Abstract

Here we describe the role of charged amino acids at the catalytic sites of *Escherichia coli* ATP synthase. There are four positively charged and four negatively charged residues in the vicinity of of *E. coli* ATP synthase catalytic sites. Positive charges are contributed by three arginine and one lysine, while negative charges are contributed by two aspartic acid and two glutamic acid residues. Replacement of arginine with a neutral amino acid has been shown to abrogate phosphate binding, while restoration of phosphate binding has been accomplished by insertion of arginine at the same or a nearby location. The number and position of positive charges plays a critical role in the proper and efficient binding of phosphate. However, a cluster of many positive charges inhibits phosphate binding. Moreover, the presence of negatively charged residues seems a requisite for the proper orientation and functioning of positively charged residues in the catalytic sites. This implies that electrostatic interactions between amino acids are an important constituent of initial phosphate binding in the catalytic sites. Significant loss of function in growth and ATPase activity assays in mutants generated through charge modulations has demonstrated that precise location and stereochemical interactions are of paramount importance.

## 1. Introduction

A typical 70 kg human generates approximately 2.0 million kg of ATP, the cell's energy currency, in a 75-year lifespan by converting food into useable energy by oxidation. ATP is generated by ATP synthase from ADP and inorganic phosphate (Pi) [1, 2]. ATP synthase is not only the essential means of cellular energy production in animals but also in plants and almost all microorganisms. ATP synthase is the final enzyme in the oxidative phosphorylation pathway and is responsible for ATP synthesis by oxidative or photophosphorylation in the membranes of bacteria, mitochondria, and chloroplasts. It is the smallest known biological nanomotor. In order to synthesize ATP, a mechanical rotation mechanism is used where subunits rotate at approximately 100 times per second. Basic [3] functional aspects of ATP synthase remain the same in both prokaryotes and eukaryotes [4].

Membrane bound F_1_F_o_ ATP synthase enzyme is structurally identical and highly conserved among different species. ATP hydrolysis and synthesis occur in the F_1_ sector, whereas proton transport occurs through the membrane embedded F_o_ [2, 5]. ATP synthesis is the result of proton gradient-driven clockwise rotation of *γ* (as viewed from the outer membrane), while ATP hydrolysis occurs from anticlockwise rotation of *γ*-sub unit. Detailed reviews of ATP synthase structure and function may be found in [6–16].

A number of diseases such as Leigh syndrome, ataxia, Batten's diseases, Alzheimer's, angiogenesis, hypertension, cancer, heart disease, mitochondrial diseases, immune deficiency, cystic fibrosis, diabetes, ulcers, and tuberculosis that affect both human and animals have been associated with ATP synthase ([1, 17] and references therein). The presence of ATP synthase on the surfaces of multiple cell types, and its involvement in a number of cellular processes, makes this enzyme an attractive molecular target in the development of treatments for numerous diseases. [18–21]. One particular way in which ATP synthase can be used as a therapeutic target is to inhibit it and thereby deprive abnormal cells of required energy leading to cell death [1, 17, 21, 22]. 

## 2. Inhibition of ATP Synthase

A wide range of natural and synthetic products are known to bind and inhibit ATP synthase [1, 17, 23, 24]. Biochemical and structural studies of ATP synthase have so far revealed about ten different inhibitor binding sites. A detailed list of known inhibitors and their actions on ATP synthase are discussed in reference [1, 17]. The inhibitory effects and the extent of inhibition on a molar scale are variable among different inhibitors. Some inhibitors prevent synthesis of ATP but not hydrolysis, or vice versa, while some are known to inhibit both synthesis and hydrolysis equally. Well-known inhibitors of ATP synthase are sodium azide (NaN3), aluminum fluoride (AlFx), scandium fluoride (ScFx), beryllium fluoride (BeFx), dicyclohexylcarbodiimide (DCCD), and 7-chloro- 4-nitrobenzo-2-oxa-1, 3-diazole (NBD-Cl) [11, 24–32]. Less well-known inhibitors of ATP synthase are peptides such as melittin, melittin-related peptide (MRP), ascaphin, aurein, caerin, dermaseptin, magainin II, and polyphenols such as resveratrol, piceatannol, quercetin, morin, and epicatechin [1, 18, 19, 21, 33–35].

The polyphenol piceatannol is one of the most portent inhibitors of ATP synthase [19, 22]. The binding site for polyphenols is at the interface of *α*-, *β*-, and *γ*-subunits of the F_1_ sector. X-ray structure shows that the following polyphenol binding pocket residues *γ*Gln274, *γ*Thr-277, *β*Ala-264, *β*Val-265, *γ*Ala-270, *γ*Thr-273, *γ*Glu-278, *γ*Gly-282, and *α*Glu-284, are highly conserved among different species and are within 4 Å of the bound polyphenol compounds. Consequently, piceatannol and other inhibitory polyphenols can form both hydrophobic and nonpolar interactions with the above residues [22, 36, 37]. We hypothesize that molecular modulation of both polyphenol-binding pocket residues and polyphenol structures may synergistically affect ATP synthase activity and provide additional clues to catalytic site function. 

The *β*DELSEED-loop of *E. coli* ATP synthase is known to be the binding site for several basic amphiphilic *α*-helical peptide inhibitors of ATP synthase. Examples are melittin, melittin-related peptide (MRP), bacterial/chloroplast ATP synthase *ε*-subunit, and SynA2 (the synthetic derivative of cytochrome oxidase). The *α*-helical basic peptide, melittin, is composed of 26 residues and is the primary component of honey bee venom (*Apis mellifera*). MRP is a 23-residue long peptide derived from frog skin (*Rana tagoi*). Both melittin and MRP are potent inhibitors of ATPase activity of *E. coli* ATP synthase [1, 21, 38, 39]. 

Most ATP synthase inhibitory peptides studied so far are from anuran (frogs) sources. These antimicrobial peptides (AMPs) are cationic, between 10 and 50 residues in length, and frequently include a C-terminal amide group [1, 40–42]. Previous mode of action studies indicate that AMPs appear to interact with negatively charged phospholipids and then insert into the bacterial cell membrane or that they may also move across the cell membrane by passive transport and there disrupt a number of cellular processes [43].

Lately, it was observed that some of the antimicrobial effects of amphibian AMPs may be through their inhibitory effects on ATP synthase [1, 21]. Melittin and other peptide inhibitors inhibit ATPase activity in a reversible and noncompetitive fashion [38, 39, 44–47]. It is hypothesized that relatively short antibacterial or anticancer cationic peptides of approximately 10–30 amino acid residues with *α*-helical secondary structure may inhibit ATP synthase through their binding to the *β*DELSEED loop. For example, lysine-induced three positive charges of dermaseptin or four positive charges of magainin II interact with the five negative charges of *β*Asp-380, *β*Glu-381, *β*Glu-384, *β*Glu-385, or *β*Asp-386 and result in the inhibition of ATPase activity. 

Of nearly 60 anuran-derived potential antimicrobial/anticancer peptides, only 13 have been tested for their inhibitory effects on ATP synthase [1, 21]. It was shown that MRP (melittin-related peptide) and MRP-amide strongly inhibited the ATPase activity of ATP synthase and that the presence of an amide group at the c-terminus of MRP caused a ~16% increase in inhibition of ATP synthase ATPase activity. Whether or not negative charges in the *β*DELSEED motif play any role in the structural stability of the catalytic sites through electrostatic interactions with site residues remains to be seen.

## 3. Structural and Functional Aspects of Charged Residues in the Catalytic Sites of ATP Synthase

Recent studies have illuminated the role of charged residues in Pi binding at the catalytic sites. Binding of inorganic phosphate (Pi) is an important step in the ATP synthase mechanism which has been extensively studied by biochemical approaches and may be directly coupled to subunit rotation [2, 11, 25, 48–53]. ATP synthase is the terminal enzyme of oxidative phosphorylation and photophosphorylation that synthesizes ATP from ADP and Pi. The energy for ATP synthesis comes from the transmembrane movement of protons down an electrochemical gradient that is generated by substrate oxidation or by light capture. As protons move through the interface between the a and c subunits in the membrane-bound F_o_-sector of the enzyme, the free energy is transduced into mechanical rotation of a group of subunits (*γε*c_10-14_) which comprise the “rotor"”. The helical coiled coil domain of the *γ*-subunit projects into the central region of the *α*_3_ *β*_3_ hexagon in the membrane extrinsic F_1_-sector. The *α*_3_ *β*_3_ hexagon contains three catalytic sites at *α*/*β* interfaces. The “Stator” subunits b_2_ and *δ* function to prevent co-rotation of *α*_3_ *β*_3_ with the rotor [6, 54–58]. In this paper we present a detailed description of the catalytic site charged amino acids, their role in Pi binding, their effects on the spatial orientation, and effect of their modulation on one another. 

**Figure 1:**
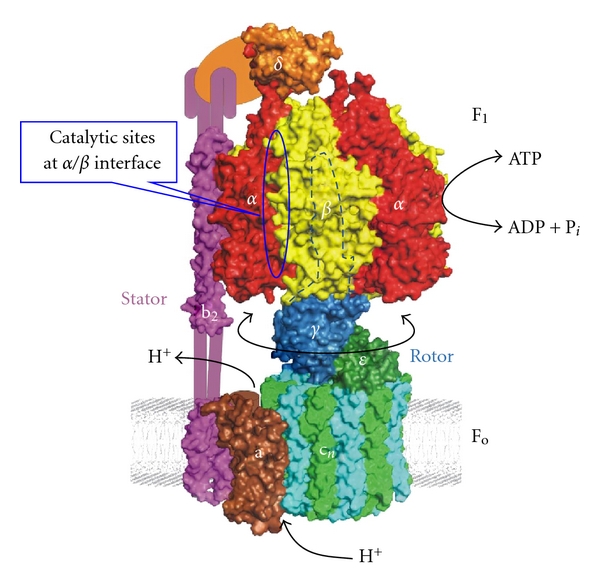
Escherichia coli ATP synthase structure: *E. coli* ATP synthase enzyme is composed of two sectors, water soluble F_1_ and membrane bound F_o_. Catalytic activity occurs at the interface of *αβ*/subunits of F_1_ sector which consists of five subunits (*α*3*β*3*γδε*) and proton conduction occurs at the Fo sector consisting of three subunits (ab_2_c). One of the catalytic binding sites is identified with circle at the interface of *α*/*β* subunits. This model of *E. coli* ATP synthase is reproduced from Weber [6] with permission; copyright Elsevier.

Figure 1 represents the simplest form of ~530 kDa* Escherichia coli* ATP synthase containing eight different subunits, namely, *α*_3_ *β*_3_ *γδε*ab_2_c_10-15_, divided into two sectors F_1_  and F_o_. F_1_ corresponds to *α*_3_ *β*_3_ *γδε* and F_o_ to ab_2_c. Overall, F_1_F_o_-ATP synthase is structurally and functionally similar among sources with only a few exceptions such as in chloroplasts, where there are two isoforms, and in mitochondria, where there are 7–9 additional subunits. ATP hydrolysis and synthesis occur on three catalytic sites in the F_1_ sector. The *γ*-subunit is comprised of three *α*-helices. Two of these helices form a coiled coil that extend into the central space of the *α*_3_ *β*_3_ hexagon. In recent nomenclature, the rotor consists of *γε*c_n_, and the stator consists of *α*_3_ *β*_3_ *δ*ab_2_. Current understanding of the F_1_F_o_ ATP synthase structure, function, catalytic mechanism, and its role in human health and disease has been thoroughly reviewed by Senior's group and others [1, 2, 7, 8, 17, 20, 59].

Based on the binding of ATP, ADP, and Pi, the three catalytic sites located on the F_1_ sector of ATP synthase are designated *β*TP, *β*DP, and *β*E by X-ray crystallographers [60, 61]. *β*E is the empty site into which Pi (inorganic phosphate) must initially bind for initiation of ATP synthesis. It has been proposed that the synthesis reaction in the three catalytic sites do not occur independently but in a consecutive manner [51]. In this “binding change mechanism”, the three catalytic sites have different affinities for nucleotides at any given moment. Each catalytic site undergoes conformational transitions that lead to the following sequence: substrate binding (ADP and Pi) → ADP phosphorylation → ATP release. Experimental observations of rotation verified the predication made by Boyer [4, 51, 62] that catalysis requires the sequential participation of the three catalytic sites, with changing affinity for substrates and products, as it proceeds through the recurring mechanism, hence the term “binding change mechanism.” Proton motive force is converted by F_o_ to a mechanical rotation of the rotor shaft, which drives conformational changes of the catalytic domains of F_1_ causing synthesis of ATP by phosphorylation of ADP. Conformational changes in the catalytic sites are connected to *γ*-subunit rotation. *γ*-Subunit rotation in isolated *α*_3_ *β*_3_ *γ* subcomplex has been observed directly by Yoshida and Kinosita with colleagues in Japan and subsequently by several other labs [12, 63–68]. The focus of this paper, the role of charged residues at the catalytic sites of *E. coli* ATP synthase, is a fundamental issue, because catalytic site charged residues are also relevant to many other ATPases, GTPases, and their potential application to nanotechnology and nanomedicine [1, 11, 18, 69].

## 4. Catalytic and Motor Function of ATP Synthase

Determination of catalytic site Pi-binding residues has allowed a better understanding of the reaction mechanism of ATP synthesis and hydrolysis, and their relationship to the mechanical rotation of the *γ*-subunit. Characterization of catalytic site-charged residues can provide essential understanding in the following possible applications (1) development of effective modulator (inhibitory/stimulatory) molecules of ATP synthase catalytic function and (2) development of catalytic site mutants for biotechnological applications. 

The analysis of charged catalytic sites residues involved in Pi binding has also helped answer the primary question of how the enzyme binds ADP and Pi rather than ATP at the catalytic sites? This is an often overlooked but crucial question in the mechanism of ATP synthesis. In active cells, the cytoplasmic concentrations of ATP and Pi are approximately in the 2–5 mM range, whereas that of ADP is at least 10–50-fold lower. Equilibrium-binding assays have established that both ADP and ATP bind to catalytic sites of purified F_1_ and detergent solubilized F_1 _F_o_ with relatively similar binding affinities [71–74]. Obviously, a specific mechanism must have evolved for selectively binding ADP into catalytic sites while simultaneously preventing ATP binding during proton driven rotation and ATP synthesis. One hypothesis is that during ATP synthesis, the proton gradient-driven rotation of subunits impels an empty catalytic site to bind Pi tightly, thus stereochemically precluding ATP binding and, therefore, selectively favoring ADP binding [7]. A second fundamental question is how does subunit rotation affect Pi binding [49–51]? It was shown that Pi binding appears to be “energy linked”, which entails a linkage to subunit rotation [56, 75, 76]. Therefore, for formulating a mechanism of ATP synthesis, it is of paramount importance to understand the features that determine Pi binding. Moreover, in the near future, it may be possible to use molecular features of Pi binding, derived from mutational and biochemical studies, in the development of potent and novel molecular modulators of ATP synthase.

## 5. Characterization of Charged Residues at the Catalytic Sites

**Figure 2:**
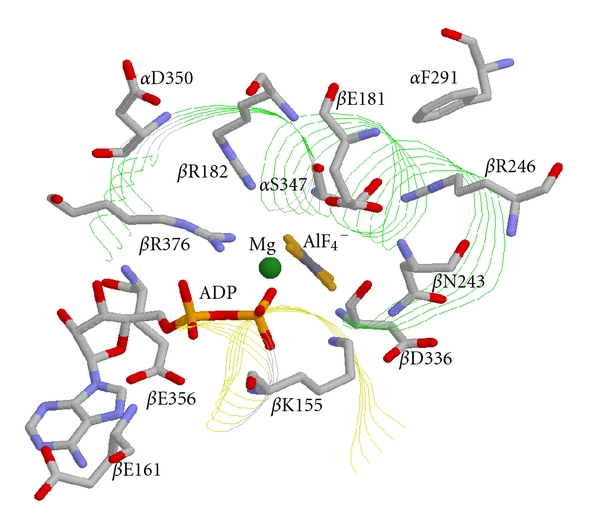
*Catalytic sites X-ray structure of ATP synthase showing spatial relationship of charged *α* and *β*-subunit residues. *The *β*DP site in the AlF_4_^−^-inhibited enzyme structure is taken from [61]. *E. coli* residue numbering is used. Four positively and four negatively charged residue in close proximity to the bound phosphate analog AlF_4_^−^ are identified. Rasmol software [70] was used to generate this figure using PDB file 1H8E [61].

X-ray structural studies and mutagenic analyses of F_1_ sector, the catalytic segment of ATP synthase, have so far identified four basic residues critical for catalysis, namely, *αβ*Lys-155, *β*Arg-182, Arg-376, and *β*Arg-246.  Figure 2 shows the spatial orientation of these amino acids in close proximity to bound phosphate analog AlF_4_^−^. *E. coli* residue numbers are used throughout.  Figure 2 also shows the spatial relationship between positive and negative charged residues.

Initial studies employed the MgATP- and MgADP-binding parameters in mutant enzymes *β*K155Q, *β*R182K, *β*R182Q, *β*R246A, *β*R246Q, *β*R246K, *α*R376K, and *α*R376Q. These studies used fluorimetric analysis with introduced *β*Trp-331 as a specific catalytic site probe, while analysis of the transition state formation was done using MgADP-fluoroaluminate and MgADP-fluoroscandium as transition state analogs [77–81]. Absent from these analyses was a direct measurement of Pi binding in the mutant enzymes. The above residues were clearly demonstrated to be involved in Pi binding with the subsequent application of Pi protection against NBD-Cl inhibition assays [11, 48, 82–85]. 

The *β*Lys-155 is part of the Walker A sequence in the catalytic sites of ATP synthase. X-ray structures of native F_1_ with bound MgAMPPNP and MgADP [5], of MgADP-BeFx inhibited F_1_ [87], of MgADP-AlF_4_- inhibited F_1_ representing the transition state [61], and of MgADP-AlF_3_ inhibited F_1_ representing the late transition state/early ground state [88] all show the *ε*-amino group of *β*Lys-155 very close (≤3 Å) to the *γ*-phosphate position. The *β*K155Q mutant lacks ATP synthesis and has very low F_1_-ATPase activity (Table 1). Previous work had shown that *β*Lys-155 plays a major role in binding MgATP, particularly at catalytic sites of high and medium nucleotide affinity, but not in binding MgADP [77]. *β*Lys-155 is also critical for transition state formation [79, 80]. The earlier hypothesis that *β*Lys-155 was important for Pi binding in ATP synthesis [2] was experimentally confirmed by Pi protection against NBD-Cl inhibition where Pi binding in the *β*E catalytic site is abolished in *β*K155Q. Therefore, residue *β*Lys-155 is involved at all stages of ATP synthesis from Pi binding, to the transition state, to MgATP formation [84]. 

*β*Arg-182 is another important positively charged phosphate-binding residue in the catalytic sites of ATP synthase [84]. Mutants *β*R182Q and *β*R182K lack ATP synthesis activity and have low F_1_-ATPase activity (Table 1). Residue *β*Arg-182 had been shown to be involved in MgATP binding at the site of highest affinity but not in MgADP binding. Transition state formation is abolished by *β*R182Q but retained in *β*R182K [78]. In this regard, it should be noted that *β*R182K F_1_ does have somewhat higher ATPase activity (Table 1). *β*Arg-182 was also hypothesized to be required for Pi binding in ATP synthesis [2], and this confirmed that both *β*R182Q and *β*R182K mutations abolished Pi binding in the *β*E site. Therefore, residue *β*Arg-182 is also involved in all stages of ATP synthesis from Pi binding through ATP formation [84]. 

The *α*Arg-376 residue of *E. coli* ATP synthase has been described as the “arginine finger” based on G-protein literature and was thought to be a required ligand for the catalytic transition state. Nonetheless, this residue was not shown to be involved in MgATP or MgADP binding despite its apparent proximity to the *γ*-phosphate of MgAMPPNP in X-ray structures [65, 81]. Movement of this residue in and out of the catalytic site was inferred and was postulated to produce the rate acceleration (“positive catalytic cooperativity”) linked to subunit rotation and full (“tri-site”) catalytic site occupancy that is a hallmark of the mechanism [2]. Significant spatial displacements of residue *α*Arg-376 have been noted in X-ray structures representing different reaction intermediates [5, 33, 61, 75, 87, 88]. Consequently, it was hypothesized that conformational freedom of this residue likely contributes to its importance in catalysis [87]. The previously hypothesized importance of this residue in catalysis [2] was confirmed by Pi protection against NBD-Cl inhibition in which Pi failed to protect *α*R376Q F_1_ from NBD-Cl inhibition. However, just as the *α*R376K mutant was able to form the transition state [81], it was also able to support Pi binding. It is nevertheless strongly impaired in both ATP synthesis and hydrolysis, which suggests that this residue has other required function(s) such as in conformational movements or in H-bonding to other side chains that are specific to Arg and not supported by Lys [11, 84]. 

**Figure 3:**
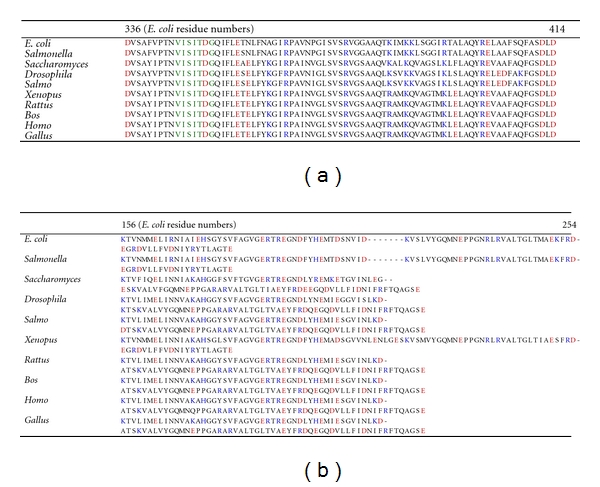
*Sequence alignment of *α* and* *β-subunit residues.* *α* and *β*-subunit amino acids from different species are aligned. Conserved positively charged residues are shown in blue color, and negatively charged residues are identified by red color. *E. coli *starting residue for *α*-subunit is *α*D336 and for *β*-subunit is *β*K155.

*β*Arg-246 is the fourth positively charged residue within the Pi-binding subdomain of catalytic sites identified in the X-ray crystallographic structure (Figure 2) that is involved in Pi binding. *β*Arg-246 is equivalent to *β*Arg-260 in mitochondrial F_1_ and is conserved among all species. Early random mutagenesis experiments revealed that mutations *β*R246H and *β*R246C impaired oxidative phosphorylation drastically and reduced ATPase activity in purified F_1_ to ~1% of wild type [89, 90]. Further work showed that these mutations caused the unisite catalysis parameter K_d_Pi to change by 4 orders of magnitude, whereas the K_d_ADP was largely unaltered by *β*R246C, and the ATP hydrolysis reaction equilibrium constant changed to favor ATP over ADP plus Pi [91]. Computer simulations have drawn attention to *β*Arg-246, where movement of the residue during rotation, conformational change of the sites [92], and a role in binding Pi in the transition state were predicted [93, 94]. Site-directed mutagenesis of *β*R246 to Gln, Lys, and Ala was used to examine the effects of each mutation on function. A variety of inhibitors and ligands known to bind or react in the catalytic sites close to the Pi-binding subdomain were utilized in combination with the mutant enzymes to establish the role of the *β*Arg-246 side chain. Substitutions of the Arg side chain to Gln (removes charge and preserves bulk), to Lys (preserves positive charge), and to Ala (removes side chain and charge) were all examined. All three substitutions severely impeded growth by oxidative phosphorylation and reduced ATPase activity of purified F_1_ to ~1% of wild type. Finally, as shown in Figure 3 Pi protection against NBD-Cl inhibition clearly demonstrated that *β*Arg-246 residue side chain is an important constituent in binding Pi and in forming the transition state [48].

Pi binding assays using Pi protection against NBD-Cl were devoid of any nucleotide and enzymes were prepared so as to have all three catalytic sites essentially empty. Therefore, the sites were in *β*E conformation. In this conformation *α*Arg-376 and *β*Arg-246 lie 2.6 and 4.0 Å from *β*Arg-182, whereas *β*Lys-155 lies 9.5, 7.3, and 6.3 Å from *α*Arg-376, *β*Arg-182, and *β*Arg-246, respectively [88]. In essence, the X-ray structure [87] showed that bound MgADP-BeFx mimicked bound MgATP. In assays of F_1_-ATPase, it was shown that wild type and *α*R376Q were fully inhibited by MgADP-BeFx, whereas *β*K155Q and *β*R182Q were fully-resistant (Z. Ahmad, and A. E. Senior, unpublished work). These results supported the hypothesis that *β*Lys-155 and *β*Arg-182 are MgATP ligands, but that *α*Arg-376 is not, and that the involvement of stringent stereochemical orientation factors plays a role in determining the functional interactions of *α*Arg-376 [11, 84]. 

The four positively charged residues form a tetrahedral structure with *β*Lys-155 at the apex and *α*Arg-376, *β*Arg-182, and *β*Arg-246 on the base [11, 25]. A potential Pi-binding pocket can readily be envisaged at the center of this tetrahedron (see Figure 2). In ATP synthesis, the *β*E site will change to the *β*ADP + Pi (“half-closed”) site in association with *γ*-rotation [2, 61]. The X-ray structure of this conformation [61] shows that the residues *α*Arg-376, *β*Lys-155, and *β*Arg-182 are each located ≤3.0 Å from the nearest oxygen atom of bound SO_4_^2-^) anion (modeling Pi), whereas *β*Arg-246 is 4.5 Å from the sulfate. Thus, as the reaction proceeds, the three residues *α*Arg-376, *β*Lys-155 and *β*Arg-182 close around the Pi and move it away from *β*Arg-246 toward the site of transition state formation [11, 12, 48, 95]. 

The above results supported the following proposed molecular mechanism for ATP synthesis [2]. Initially, substrate Pi binds in the *β*E catalytic site using four basic residues as ligands, namely, *α*Arg-376, *β*Arg-182, *β*Lys-155, and *β*Arg-246 [11, 25, 48, 82–84, 86]. After binding of MgADP (in which these four residues are not involved), the catalytic transition state forms using *α*Arg-376, *β*Arg-182, and *β*Lys-155 as direct ligands. Upon formation of MgATP, *α*Arg-376 withdraws and no longer interacts, whereas *β*Lys-155 and *β*Arg-182 are still bound to the *γ*-phosphate. MgATP is released to the medium with the breaking of these bonds. 

Historically, many attempts to measure Pi binding in purified *E. coli* F_1_ using [^32^P] Pi [50] or by competition with ATP or AMP-PNP in fluorescence assays of nucleotide binding [72, 96] failed to detect appreciable Pi binding at physiological Pi concentration. An assay devised by Perez et al. [97] in which the protection afforded by Pi against the inhibition of ATPase activity was induced by covalent reaction with 7-chloro-4-nitrobenzo-2-oxa-1, 3,-diazole (NBD-Cl) provided a measure of Pi binding. Orriss et al. [98] showed by X-ray crystallography that the covalent adduct formed by NBD-Cl is specifically in the *β*E catalytic site. Hence, protection afforded by Pi indicates that binding of Pi occurs at the *β*E catalytic site. By modifying the above assay for use with *E. coli* purified F_1_ or membrane bound F_1 _F_o_, further studies have to date investigated the relationship between Pi binding and catalysis for eight residues, namely *α*Phe-291, *α*Ser-347, *α*Gly-351, *α*Arg-376, *β*Lys-155, *β*Arg-182, *β*Asn-243, and *β*Arg-246. It was shown that the five residues *α*Ser-347, *α*Arg-376, *β*Lys-155, *β*Arg-182, and *β*Arg-246 were grouped in a tetrahedral relationship,and are involved in Pi binding. The other three residues *α*Phe-291, *α*Gly-351, and *β*Asn-243 are not involved in Pi binding [11, 25, 48, 82–84, 86]. In consequence, the presence of positively charged residues in the catalytic site explains the preferential binding of ADP over ATP. 

It may be noted that [^32^P]Pi binding was detected with a Kd(Pi) in the range of 0.1 mM using an alternative, pressure ultrafiltration method, and this result is consistent with data obtained from the NBD-Cl protection assay [99]. It is apparent that Pi dissociates more rapidly from *E. coli* F_1_ than it does from mitochondrial F_1_, undesirably, rendering the convenient centrifuge assay incompatible with the *E. coli* enzyme.

The story goes on with the presence of many charged/uncharged residues in close proximity to the Pi binding subdomain in the catalytic sites. These residues have been shown to exert either a direct or an indirect role in Pi binding. One of these residues is the charged *α*Asp-350 of the VISIT-DG sequence. *α*Asp-350 is part of the *α*-subunit VISIT-DG sequence, which is a highly conserved motif in this enzyme [25]. VISIT-DG sequence residues are of special interest in general and negatively charged *α*Asp-350 in particular, because of the close proximity to the known positively charged phosphate-binding residues. *α*Asp-350 is ~3 Å from *α*Arg-376. It would be imperative to understand three specific aspects of *α*Asp-350 residue. First, is residue Asp-350 directly or indirectly involved in phosphate binding through *α*Arg-376? Second, is *α*Asp-350 important for function through its role in maintaining the structural integrity of the Pi binding subdomain but not involved in Pi binding *per se*? Third, is the carboxyl side chain of *α*Asp-350 involved in the transition state at the catalytic site? Our hypothesis is that *α*Asp-350 interacts electrostatically with *α*R376. Such an interaction may provide proper orientation of *α*Arg-376 side chain towards Pi.

## 6. Modulation of Charge in the Catalytic Sites

**Figure 4:**
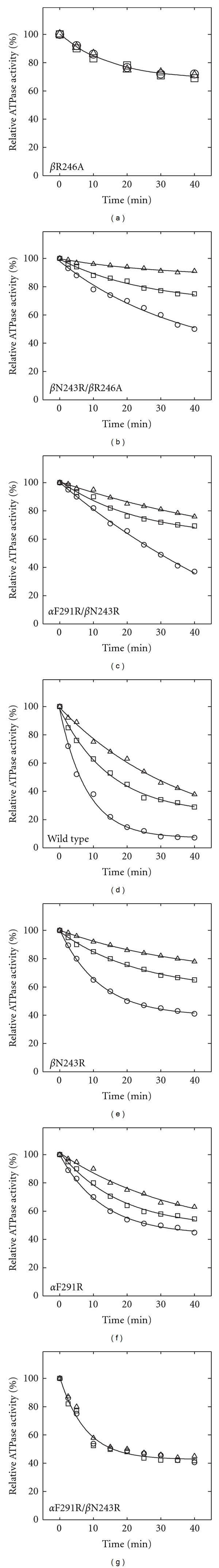
*Compensatory effect inserted arginine residue.* Membranes were preincubated with Pi at zero, 2.5 or 10 mM concentration as shown, for 60 min at 23°C. Then, NBD-Cl (125 *μ*M) was added and aliquots withdrawn for assay at time intervals as shown. ATPase activity remaining is plotted against time of incubation with NBD-Cl. ○, no Pi added; □, 2.5 mM Pi; Δ, 10 mM Pi. Data taken from [83].

**Figure 5:**
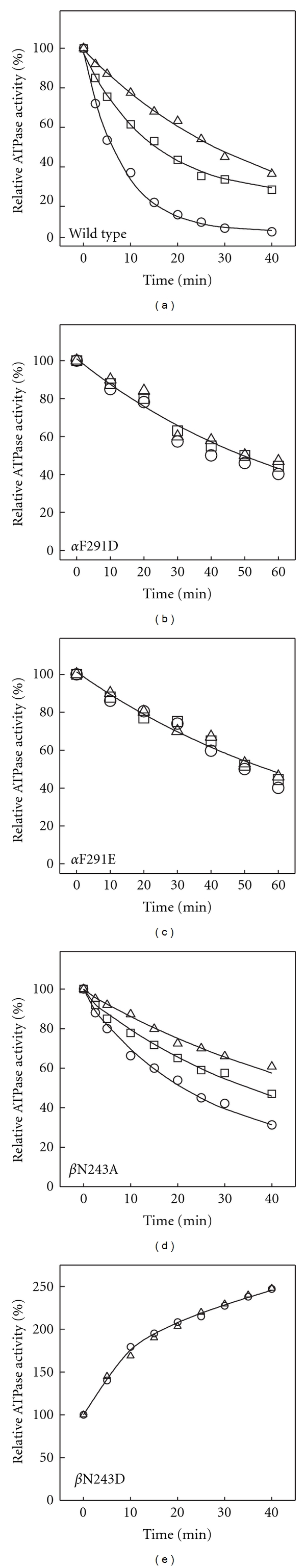
*Loss of Pi protection from inactivation by NBD-Cl inhibition with inserted negative charge. *Membranes were preincubated with Pi at zero, 2.5 or 10 mM concentration as shown, for 60 min at 23°C. Then, NBD-Cl (125 *μ*M) was added and aliquots withdrawn for assay at time intervals as shown. ATPase activity remaining is plotted against time of incubation with NBD-Cl. ○, no Pi added; □, 2.5 mM Pi; Δ, 10 mM Pi. Data taken from [82, 86].

Understanding the role of charged residues and the effects of modifying them is important in understanding the molecular mechanism of ATP synthesis. NBD-Cl inactivation assay described earlier have shown that positively charged residues are functionally essential for Pi binding in the *β*E catalytic site of *E. coli* ATP synthase [11, 48, 83, 84]. The introduction of charged residues in place of uncharged residues in the vicinity of catalytically important residues has been shown to affect Pi binding by resulting in a loss or gain of ATPase activity [82, 83, 86]. Earlier work [82, 83] indicated that the introduction of negative charge in the Pi-binding pocket in the form of *β*N243D close to *β*Arg-246 prevented Pi binding (Figure 5). Also, introduction of positive charge in the form of *β*N243R restored Pi binding in *β*R246A mutants. Similarly, the introduction of negative or positive charge in the form of *α*F291D/E/R with *β*R246 or *β*R246A resulted in a loss or gain of Pi binding (Figures 4 and 5) [83, 86]. These results suggested that modulation of charge in the Pi binding site could be used to understand the molecular mechanism of Pi binding. It is established that Arg residues occur particularly commonly in the Pi binding sites of proteins [100]. Therefore, varying the number of Arg residues in the Pi binding site of ATP synthase can be an instructive approach. 

Residue *β*Asn-243 lies 3.2 Å from *β*Arg-246 in both AlF_3_ and SO_4_^2−^)-containing catalytic sites (nearest atom distances quoted) [83]. One experimental approach used was to introduce the mutation *β*N243R in a wild-type background (with *β*Arg-246) and in presence of the *β*R246A mutation. Residue *α*Phe-291, located at the end of the Pi-binding pocket across the catalytic *α*/*β* interface, with its side-chain pointing toward the bound Pi analogs, is also a suitable location for introducing a new Arg. This residue lies at a distance of 3.2 Å from *β*Arg-246 in the AlF_3_-containing catalytic site and 7.5 Å in the SO_4_^2−^-containing catalytic site [61, 83, 88]. Arginine was introduced in the form of an *α*F291R mutation in the wild-type background and in the presence of the *β*R246A mutation. The actual distances of residues *β*Arg-246, *β*Asn-243 and *α*Phe-291 were obtained from bound AlF_3_ and SO_4_^2−^ as determined by X-ray crystallography [61, 88], while speculative distances (in brackets) were calculated for mutant residues *β*Ala-246, *β*Arg-243 and *α*Arg-291 using the “Deep View Swiss-Pdb Viewer” [101]. Apparently, mutations placed extra positive charge relatively close to Pi, and the *β*Ala-246 mutation left a relatively large space into which a new Arg fits nicely. 

## 7. Synergistic Stereochemical Interactions at the Catalytic Sites

The introduction of one or two extra positively charged Arg residues in the wild-type background at either *β*-243 or *α*-291, or both, has proven to inhibit Pi binding (Figure 4). Introduction of a new Arg at *β*-243 or *α*-291 in the *β*R246A background provided a significant compensatory effect on ATPase. ATP-driven proton pumping was also reinstated in the case of the *α*F291R/*β*R246A mutant. But these new arginines did not restore function to full normal [83]. 

The *β*R246A mutant did not show Pi binding, but both *β*N243R and *α*F291R mutations “rescued” Pi binding in combination with *β*Ala-246 (Figure 4). Since neither *β*Arg-243 nor *α*Phe-291 could be expected to assume the exact same stereochemical interactions that *β*Arg-246 achieves, electrostatic interaction appears to be a significant factor. Presence of at least one positive charge at this general location is a requisite determinant of initial Pi binding in the catalytic site *β*E. *β*N243R or *α*F291R in the wild-type background (representing one extra positive charge) did not prevent Pi binding, but the combination of *α*F291R/*β*N243R (two extra charges) abrogated Pi binding (Figure 4). Presumably the local concentration of charge in the latter becomes too disruptive and distorts the Pi-binding site [83]. 

A similar pattern of effects has been reported when transition state stabilization was assessed by assaying inhibition of ATPase activity by the transition state analogs MgADP-fluoroaluminate and MgADP-fluoroscandium. Previously, it was shown that [48] that both inhibitors are potent against wild-type ATP synthase but that each inhibit *β*R246A mutant only to small extent, which indicates that *β*Arg-246 is intimately involved in transition state stabilization. It was found that either mutant residue *β*Arg-243 or *α*Arg-291 partly “rescued” transition state stabilization when present with *β*Ala-246 [83]. Raising the number of positively charged residues to two (*β*N243R and *α*F291R mutants in wild-type background) had an adverse effect as reflected by a lesser inhibition of ATPase activity. Raising the number of local positive charges to three reduced transition state stabilization right back to where it was in *β*R246A. Interestingly, even in the best cases among the mutants (*β*N243R/*β*R246A or *α*F291R/*β*R246A) transition state stabilization was incomplete as compared to wild-type, which suggests the importance of stereochemical interactions [83].

In summary, all the results showed that Pi binding is notably affected by the local positive charges in catalytic site *β*E of ATP synthase. Positive charge in the vicinity of the natural *β*Arg-246 is important because its removal in *β*R246A mutant can be compensated partially by introduction of one Arg at either *β*-243 or *α*-291. Thus, electrostatic interaction is an important determinant of Pi binding. The presence of two arginines by introduction of either *β*Arg-243 or *α*Arg-291 in the presence of *β*Arg-246 does not prevent Pi binding, but the presence of all three arginines eliminates Pi binding. Effects on transition state stabilization followed a parallel pattern, but the restoration of Pi binding in *β*E catalytic sites by charge compensation was not sufficient by itself to restore full function [83].

## Figures and Tables

**Table 1:** ATPase activity of *E. coli* membrane bound or purified F_1_ enzymes.

Mutation ^a^	ATPase activity *μ*mol/min/mg
Wild-type	28.0 (42.0)
Null	0.0013
*β*K155Q	(0.023)
*β*R182K	(0.250)
*β*R182Q	(0.020)
*α*R376K	(0.120)
*α*R376Q	(0.025)
*α*F291D	0.07
*α*F291E	0.09
*β*N243A	0.95
*β*N243D	0.033
*β*R246A	0.050 (0.25)
*β*R246K	(0.27)
*β*R246Q	(0.27)
*β*N243R	0.023
*β*N243R/*β*R246A	0.016
*α*F291R	0.035
*α*F291R/*β*R246A	0.52
*α*F291R/*β*N243R	0.028

^a^Wild-type, pBWU13.4/DK8; Null, pUC118/DK8. All mutants were expressed with the *β*Y331W mutation also present, which does not significantly affect growth. Data are means of four to six experiments each.  ^b^Measured at 37°C and expressed as *μ*mol ATP hydrolyzed/min/mg membrane protein. Each individual experimental point is itself the mean of duplicate assay tubes. Data in parentheses is from purified F_1_ ATP synthase. Data taken from [48, 82–84, 86].
